# Low serum TSH levels are associated with low values of fat-free mass and body cell mass in the elderly

**DOI:** 10.1038/s41598-021-90178-7

**Published:** 2021-05-18

**Authors:** Till Ittermann, Marcello R. P. Markus, Martin Bahls, Stephan B. Felix, Antje Steveling, Matthias Nauck, Henry Völzke, Marcus Dörr

**Affiliations:** 1 Institute for Community Medicine, University Medicine Greifswald, Greifswald, Germany; 2 Department of Internal Medicine B – Cardiology, Intensive Care, Pulmonary Medicine and Infectious Diseases, University Medicine Greifswald, Greifswald, Germany; 3 Institute for Clinical Chemistry and Laboratory Medicine, University Medicine Greifswald, Greifswald, Germany; 4 German Centre for Cardiovascular Research (DZHK), Greifswald, Germany; 5 DZD (German Center for Diabetes Research), Greifswald, Germany; 6 Department of Medicine A, University Medicine Greifswald, Greifswald, Germany

**Keywords:** Biomarkers, Endocrinology, Medical research, Risk factors

## Abstract

Previous studies on the association between thyroid function and body composition are conflicting and showed strong differences across age groups. Our aim was to clarify age-specific associations of serum thyroid-stimulating hormone (TSH) levels with markers of body composition including body mass index (BMI), waist circumference, fat mass (FM), fat-free mass (FFM) and body cell mass (BCM). We used data from two independent population-based cohorts within the framework of the Study of Health in Pomerania. The study population included 5656 individuals aged 20 to 90 years. Markers of body composition were measured by bioelectrical impedance analysis. Serum TSH levels were significantly positively associated with BMI (β = 0.16; 95% confidence interval [CI]: 0.06 to 0.27), waist circumference (β = 0.35; 95% CI: 0.08 to 0.62) and FM (β = 0.32; 95% CI: 0.12 to 0.52), but not with FFM and BCM. Interaction analysis revealed positive associations of serum TSH levels with BMI, waist circumference, FM, FFM and BCM in individuals older than 60 years, while no such associations were observed in younger individuals. We demonstrated that lower serum TSH levels were accompanied with lower values of BMI, waist circumference, FM, FFM, and BCM in the elderly, while no such associations were observed in younger individuals.

## Introduction

The prevalence of obesity has been dramatically increased during the last decades reaching epidemic proportions^1^. This trend is accompanied by a higher prevalence of cardiovascular events, cardiovascular mortality, type 2 diabetes mellitus, and cardiovascular risk factors^2–6^. Thus, it is important to minimize risk factors involved in the pathogenesis of obesity. With respect to thyroid dysfunction, several cross-sectional population-based studies in adults as well as in children demonstrated significant associations of high thyroid-stimulating hormone (TSH) with body mass index (BMI) and waist circumference^7–12^. Likewise, increasing serum TSH levels in the reference range were associated with a higher risk to be obese in cross-sectional population-based studies^8,10^. Longitudinal studies, however, failed to show positive associations of serum TSH levels at baseline with changes in body weight or waist circumference^8,11^.

To understand effects of thyroid dysfunction on body composition, it is important to not just look at the global obesity marker BMI but also at specific markers of body composition including fat mass (FM), fat-free mass (FFM) and body cell mass (BCM). While there are many studies investigating associations of thyroid function with BMI, fewer studies researched potential associations of thyroid dysfunction with muscle or lean mass. In a cross-sectional, population-based study with 946 males aged 25 to 45 years no significant association between serum TSH levels and lean mass was observed^13^.

All other studies on such potential associations were conducted in the elderly and revealed conflicting results^14–17^. In a study with 408 women ≥ 65 years old serum TSH levels at baseline were associated with low appendicular muscle mass after a mean follow-up time of 4.3 years ^16^, whereas in a cross-sectional population-based study with 6974 individuals ≥ 50 years serum TSH levels were not associated with muscle mass determined by bioelectrical impedance analysis (BIA)^17^. Furthermore, one cross sectional^15^ and one longitudinal^14^ population-based study failed to demonstrate significant associations between subclinical hypothyroidism and FFM. Against this background the aim of the present study was to clarify age-specific associations of serum TSH levels with markers of body composition including BMI, waist circumference, FM, FFM and BCM. According to a recent meta-analysis thyroid hormone levels seemed to be more strongly related with clinical parameters than TSH^18^. Thus, we further considered structural parameters of the feedback loop in our analyses ^19^. For this, we use data from two cross-sectional population-based studies, where FM, FFM and BCM were determined by bioelectrical impedance analysis (BIA).

## Results

There were 5,056 individuals with serum TSH levels within the reference range, 435 individuals with low and 165 individuals with high TSH levels (Table 1). Individuals in the low TSH group were more often males and in median older than individuals with serum TSH levels within the reference range. Markers of body composition did not differ considerably between groups of low TSH and TSH within the reference range. Individuals with high TSH were less often males and in median younger than individuals with serum TSH levels within the reference range. Markers of body composition, particularly FFM and BCM, were lower in the high TSH group compared to individuals with serum TSH levels within the reference range.

**Table 1:** Characteristics of the study population.

	TSH in reference range (n = 5056)	Low TSH (n = 435)	High TSH (n = 165)
Age; years	52 (41; 64)	62 (50; 71)	47 (34; 62)
Males	2612 (51.7%)	240 (55.2%)	60 (35.4%)
**Smoking status**			
Former	1899 (37.6%)	183 (42.1%)	59 (35.8%)
Current	1319 (26.1%)	103 (23.7%)	50 (30.3%)
Body mass index; kg/m^2^	27.4 (24.5; 30.8)	27.7 (25.0; 31.3)	26.5 (22.5; 30.4)
Waist circumference; cm	91 (81; 101)	93 (83; 104)	87 (76; 98)
Fat mass; kg	22.1 (17.2; 28.4)	22.3 (17.1; 28.1)	21.0 (16.4; 28.7)
Fat-free mass; kg	56.2 (47.1; 66.8)	57.4 (47.7; 66.3)	51.4 (45.4; 63.2)
Body cell mass; kg	29.0 (23.9; 36.0)	28.9 (23.3; 34.7)	26.2 (23.1; 33.6)
Systolic blood pressure; mmHg	129 (116; 141)	132 (119; 145)	123 (113; 138)
Diastolic blood pressure; mmHg	78 (71; 85)	77 (70; 85)	77 (69; 83)
SPINA-GT	3.35 (2.66; 4.25)	9.04 (7.65; 12.48)	1.54 (1.36; 1.78)
SPINA-GD	32.8 (29.6; 36.2)	31.7 (28.3; 35.3)	35.0 (32.0; 39.7)
Jostel’s TSH index	1.96 (1.62; 2.28)	0.81 (0.47; 1.06)	3.10 (2.91; 3.39)

Continuous data is expressed as median, 25th, and 75th percentile; categorical data as absolute numbers and %

*SPINA-GT* thyroid's secretory capacity.

*SPINA-GD* sum activity of step-up deiodinases.

In multivariable linear regression adjusted for age, sex, smoking status and study, serum TSH levels used as continuous variable were significantly positively associated with BMI (β = 0.16; 95% confidence interval [CI] = 0.06 to 0.27), waist circumference (β = 0.35; 95% CI = 0.08 to 0.62) and FM (β = 0.32; 95% CI = 0.12 to 0.52), but not with FFM and BCM (Table 2). However, individuals with high or low TSH had no significant different mean values of BMI, waist circumference, FM, FFM and BCM than individuals with serum TSH levels in the reference range. With one exception, we observed similar results when indexing FM, FFM, and BCM to squared height (Supplementary Table 1). High TSH was significantly inversely associated with the BCM index. After exclusion of individuals with high or low TSH, serum TSH levels in the reference range were positively associated with BMI (β = 0.47; 95% CI = 0.25 to 0.69), waist circumference (β = 1.01; 95% CI = 0.45 to 1.56), and FM (β = 0.90; 95% CI = 0.48 to 1.31) but not with FFM and BCM (Table 3).

**Table 2:** Associations between serum TSH levels and markers of body composition.

	TSH; mIU/L β (95%-CI)	0.49 ≤ TSH < 3.29 β (95%-CI)	TSH < 0.49 β (95%-CI)	TSH ≥ 3.29 β (95%-CI)
Body mass index; kg/m^2^	0.16 (0.06; 0.27)*	Reference	− 0.31 (− 0.76; 0.16)	− 0.15 (− 0.88; 0.59)
Waist circumference; cm	0.35 (0.08; 0.62)*	Reference	− 0.65 (− 1.81; 0.52)	− 0.17 (− 1.99; 1.66)
Fat mass; kg	0.32 (0.12; 0.52)*	Reference	− 0.47 (− 1.34; 0.41)	0.12 (− 1.24; 1.49)
Fat-free mass; kg	0.11 (− 0.06; 0.28)	Reference	− 0.28 (− 1.01; 0.44)	− 0.25 (− 1.39; 0.88)
Body cell mass; kg	0.02 (− 0.08; 0.12)	Reference	− 0.32 (− 0.76; 0.12)	− 0.54 (− 1.23; 0.14)

Association between TSH and markers of body composition were analyzed by linear regression models adjusted for age, sex, smoking status and study. We calculated two models for each outcome – the first with continuous TSH and the second with TSH categorized into three groups with 0.49 ≤ TSH < 3.29 as reference category.

CI confidence interval.

*p < 0.05.

**Table 3:** Associations between serum TSH levels in the reference range and markers of body composition.

	TSH in the reference range; mIU/L β (95%-CI)
Body mass index; kg/m^2^	0.47 (0.25; 0.69)*
Waist circumference; cm	1.01 (0.45; 1.56)*
Fat mass; kg	0.90 (0.48; 1.31)*
Fat-free mass; kg	0.33 (− 0.01; 0.68)
Body cell mass (BCM); kg	0.05 (− 0.15; 0.26)

Association between TSH in the reference range and markers of body composition were analyzed by linear regression models adjusted for age, sex, smoking status and study. Individuals with serum TSH levels < 0.49 mIU/L or TSH ≥ 3.29 mIU/L were excluded from these analyses.

CI confidence interval.

*p < 0.05.

**Figure 1:**
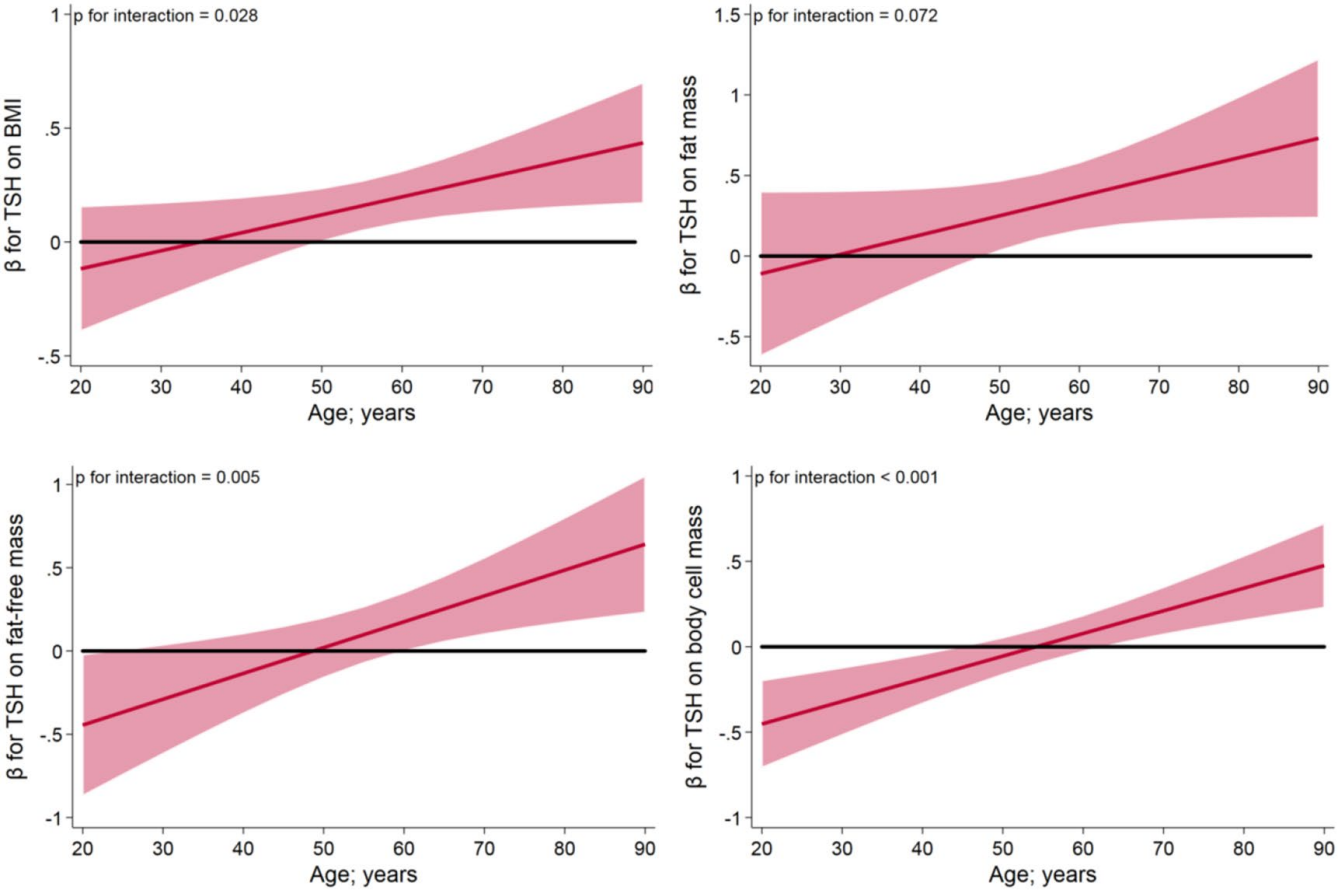
Significant interactions of TSH with age on markers of body composition. Results are displayed as age-specific β coefficients and 95% confidence intervals for TSH on **a** body mass index, **b** fat mass, **c** fat-free mass and **d** body cell mass.

There were no significant interactions of TSH with sex on markers of body composition, while interactions of TSH with age were significantly associated with all outcomes (Table 4). Interaction analyses revealed that serum TSH levels were significantly positively associated with BMI and FM in individuals older than 50 years but not in individuals younger than 50 years (Fig. 1). Similar results were observed for the effect modification of age on the association between TSH and waist circumference. Positive associations of serum TSH levels with FFM and BCM were observed in the elderly (≥ 60 years; Fig. 1), whereas in individuals < 60 years serum TSH levels were not significantly associated with FFM. In individuals younger than 40 years serum TSH levels were inversely associated with BCM. Figure 1 shows age-specific β coefficients for serum TSH levels on markers of body composition together with their 95% confidence intervals for based on the interaction models.

**Table 4:** Interactions of serum TSH levels with age and sex on markers of body composition.

	Interaction age and TSH p	Interaction sex and TSH p
Body mass index; kg/m^2^	0.028	0.966
Waist circumference; cm	0.056	0.902
Fat mass; kg	0.072	0.830
Fat-free mass; kg	0.005	0.741
Body cell mass (BCM); kg	< 0.001	0.535

*p* values derived from linear regression models with TSH, age, sex, smoking status, study and the respective interaction term.

SPINA-GD decreased significantly by age (β =  − 0.09; 95% confidence interval:  − 0.10 to  − 0.08; *p* < 0.001). We observed significant positive associations of SPINA-GD with BMI, waist circumference, FFM, and BCM but not with FM (Table 5). On the other hand, we could not show significant associations of SPINA-GT with any marker of body composition. Jostel’s TSH was positively associated with BMI, waist circumference, and FM but not with FFM. Furthermore, Jostel’s TSH was inversely associated with BCM.

**Table 5:** Associations of thyroid's secretory capacity (SPINA-GT), the sum activity of step-up deiodinases (SPINA-GD) and Jostel’s TSH index with markers of body composition.

	SPINA-GT β (95%-CI)	SPINA-GD β (95%-CI)	Jostel’s TSH index β (95%-CI)
Body mass index; kg/m^2^	− 0.001 (− 0.004; 0.002)	0.035 (0.010; 0.061)*	0.37 (0.18; 0.57)*
Waist circumference; cm	0.000 (− 0.007; 0.008)	0.090 (0.026; 0.153)*	0.80 (0.31; 1.28)*
Fat mass; kg	− 0.000 (− 0.006; 0.006)	0.045 (− 0.002; 0.093)	0.89 (0.52; 1.25)*
Fat-free mass; kg	− 0.001 (− 0.006; 0.004)	0.058 (0.018; 0.098)*	− 0.05 (− 0.36; 0.25)
Body cell mass; kg	− 0.002 (− 0.005; 0.001)	0.107 (0.084; 0.131)*	− 0.19 (− 0.37; − 0.11)*

Associations between thyroid-related exposures and markers of body composition were analyzed by linear regression models adjusted for age, sex, smoking status and study.

CI confidence interval.

*p < 0.05.

**Figure 2:**
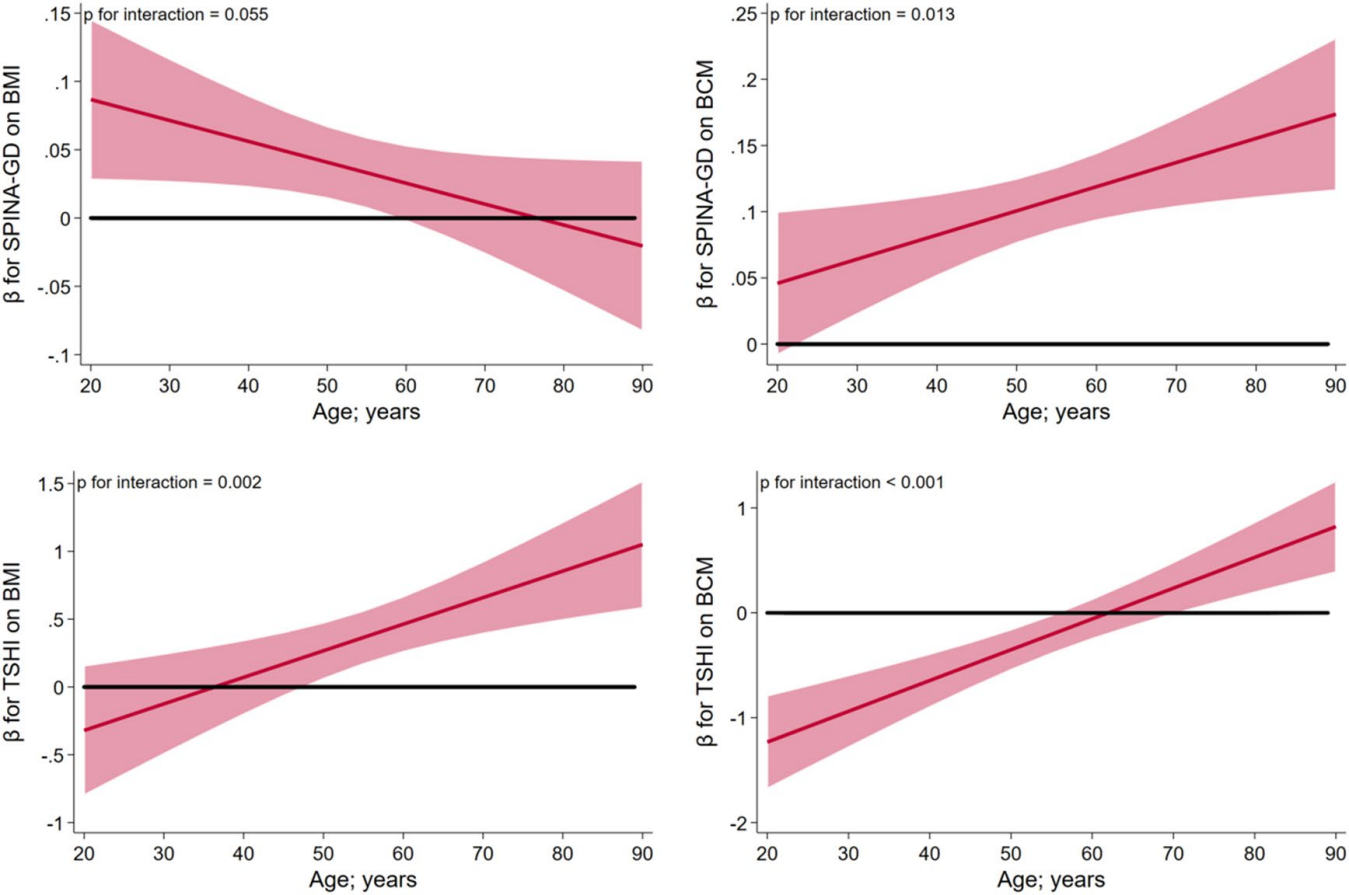
Interactions of SPINA-GD and Jostel’s TSH (TSHI) with age on body mass index (BMI) and body cell mass. Results are displayed as age-specific β coefficients and 95%-confidence intervals.

For SPINA-GD we observed significant interactions with age on the outcomes BMI and BCM (Supplementary Table 2). SPINA-GD was positively associated with BMI in individuals younger than 60 years, while no such association was observed in the elderly (Fig. 2). SPINA-GD was positively associated with BCM over the full age range, but associations were stronger in the elderly. For Jostel’s TSH we observed significant interactions with age for all outcomes except FM. For BMI and waist circumference, we observed positive associations of Jostel’s TSH in the elderly, while no significant associations were observed in younger individuals. Furthermore, Jostel’s TSH was positively associated with FFM and BCM in the elderly, while in younger individuals these associations were inverse.

## Discussion

In the present analysis we investigated associations of serum TSH levels with body composition in two large population-based studies under exclusion of individuals taking thyroid medication. We found positive associations of serum TSH levels with BMI, waist circumference, FM and FFM in the elderly, while in younger individuals only an inverse association with BCM was observed. These positive associations may be either explained by higher levels of BMI, waist circumference, FM, and FFM in individuals with high TSH levels or to lower levels of these markers in individuals with low TSH levels.

The positive associations of TSH with BMI and waist circumference observed in our study are in line with results from a previous study including cross-sectional data of five population-based studies from Germany, Netherlands and Denmark^8^. Likewise, further cross-sectional studies from Norway^10^, Denmark^12^ and USA^11^ confirmed this finding. Longitudinal studies, however, reported conflicting results. While most of the studies did not demonstrate significant associations between serum TSH levels at baseline and changes in BMI^11,14,20^, one study, which used data from four European population-based studies, reported an inverse association between serum TSH levels at baseline and five-year-changes in BMI^8^. This finding may be explained by the fact that in three of the four included studies participants were informed about their TSH level, which may have resulted in a reduction of serum TSH levels due to levothyroxine treatment in individuals with high TSH levels. This reduction in TSH levels may have led to a reduction in body weight. In agreement with this assumption several population-based studies demonstrated that a decline in serum TSH levels was associated with decreased body weight^7,8,20^. These findings argue for only a short-term effect of serum TSH levels on the development of body weight.

Until the middle of 1990ies, when an iodine fortification program was initiated, Germany was a region of mild to moderate iodine deficiency^21,22^. As a consequence, TSH levels are on average lower in the elderly than in younger individuals of our study region, because the elderly suffered longer from iodine deficiency^23^. Likewise, in our population low TSH values are more frequently found than high TSH values. Thus, the observed positive association of serum TSH levels with FFM and BCM in the elderly may be related to a reduced FFM in individuals with low TSH. A reduced FFM is the main contributing factor for sarcopenia, a condition which is defined as an age-related loss of muscles^24^. In contrast to our findings, a previous study in 6974 individuals older than 50 years found no significant associations of subclinical hyperthyroidism with low FFM and sarcopenia^17^. In that study, however, the prevalence of subclinical hyperthyroidism was only 0.7%, which may explain the discrepant findings to our study, in which the prevalence of low TSH levels was 7.7%.

Such an association is plausible because hyperthyroidism has several metabolic effects such as an increase of O2 consumption, thermogenesis and increased lipid mobilization^25,26^. These factors contribute to a loss of weight ^26^ particularly because of a loss in FFM^27^. In the younger individuals of our study we did not observe a significant association of TSH with FFM, which agrees with a previous study conducted in 941 males aged 25–45 years^13^. This lack of an association may be because younger individuals were less affected by the previous iodine deficiency resulting in a much lower number of individuals with low TSH values.

Of the four studies, which investigated associations between thyroid function and FFM in the elderly, two were focusing on the effects of subclinical hypothyroidism on FFM measured by DXA in iodine replete regions^14,15^. In that studies no significant associations of subclinical hypothyroidism with FFM were demonstrated. These findings are in agreement with a large population-based study from Brazil, in which no association between serum TSH levels and FFM determined by BIA was observed^17^. Another Brazilian study, however, demonstrated a significant positive association of serum TSH levels at baseline with a low muscle mass index (as defined as muscle mass in arms and legs divided through BMI) at follow-up in 408 women older than 65 years^16^. The different findings of that study^16^ to our and other studies^14,15,17^ may be explained by different study populations (only women included), different histories of iodine supply and particularly by different outcome definitions. While our and the other studies were focusing on total FFM^14,15,17^, Machado et al. looked at appendicular muscle mass, an outcome which may be more tightly related to muscle strength than to total FFM. In line with this assumption previous studies showed significant associations between high TSH values and muscle strength determined by a handgrip strength test^17,28^.

Looking at further markers of the thyroid feedback loop we observed positive associations of Jostel’s TSH with FFM and BCM in the elderly but not in younger individuals. This pattern is similar to the one observed for serum TSH levels in our study and may indicate that independent of the feedback loop a decreased production of TSH in the pituitary gland may result in a reduction of FFM in the elderly. Furthermore, we showed associations of lower 5’-deiodinase activity as assessed by SPINA-GD with lower BCM, which was more pronounced in the elderly. Since SPINA-GD was lower in the elderly one may speculate that due to ageing the conversion from fT4 to fT3 is reduced, which inhibits the metabolic effects of thyroid hormones and, thus, also reduces FFM.

Strengths of our study are its population-based design and the large number of individuals covering a wide adult age range of 20 to 90 years. Limitations arise from the cross-sectional design of our study not allowing to draw causal inference. Furthermore, in our study body composition was assessed by BIA rather than by DXA, which is the gold-standard method for determination of FFM but due to German jurisdiction it is not possible to conduct DXA in population-based German studies because of the radiation.

We demonstrated that lower serum TSH levels were accompanied with lower values of BMI, waist circumference, FM, FFM, and BCM in the elderly, while no such associations were observed in younger individuals. Thus, our results may indicate that treatment of older individuals with low TSH levels could prevent harmful effects of sarcopenia. Further, particularly longitudinal studies are needed to verify our findings.

## Material and methods

Analyses are based on data from two independent cohorts of the Study of Health in Pomerania (SHIP), conducted in the Northeast Germany^29^. For the baseline examination of the first SHIP cohort (SHIP-0) 6267 eligible subjects were randomly selected from population registries, of which 4,308 individuals were examined between 1997 and 2001 (response 68.8%). In the present analyses we used data from the second follow-up of SHIP-0 (SHIP-2), in which 2333 individuals aged 30–93 years were examined between 2008 and 2012. In parallel to SHIP-2, a second independent cohort (SHIP-Trend-0) was established in the same study region. For this study, 8,800 eligible subjects were randomly chosen from population registries and 4420 individuals aged 20–84 years participated (net response 50.1%). From the 6753 participants in SHIP-0 and SHIP-Trend, we excluded 763 individuals, who took thyroid medication. Furthermore, we excluded 334 individuals with missing data in any of the considered outcomes, exposures or confounders resulting in a study population of 5656 individuals.

Information on medication intake and smoking status was gathered by computer-assisted personal interviews. All participants were asked to bring all medications taken 7 days prior to the time of examination. Medication data were obtained online using the IDOM program (online drug-database leaded medication assessment) and categorized according to the Anatomical Therapeutical Chemical (ATC) classification index. Thyroid medication was defined by the ATC code H03. Smokers were categorized into three categories (lifetime non-smokers, former smokers, and current smokers). Height and weight were measured for the calculation of the BMI = weight (kg) / height^2^ (m^2^). Waist circumference was measured to the nearest 0.1 cm using an inelastic tape midway between the lower rib margin and the iliac crest in the horizontal plane with the subject standing comfortably with weight distributed evenly on both feet. Systolic and diastolic blood pressures were measured three times after an initial five-minute rest period on the right arm of seated individuals using a digital blood pressure monitor. Measurements were separated by three-minute intervals. The mean of the second and third measurements was calculated and used for the present analyses.

FM, FFM, and BCM were measured by BIA using a multifrequency Nutriguard‐M device (Data Input, Pöcking, Germany) and the NUTRI4 software (Data Input, Pöcking, Germany) in participants without pacemakers. The electrodes were placed on hand, wrist, ankle, and foot. The test frequency was measured at 5, 50, and 100 kHz following the manufacturer's instructions^30^. Indices were calculated in kg/m^2^ by dividing FM, FFM, and BCM by squared body height.

Non-fasting blood samples were taken between 07:00 a.m. and 04:00 p.m.. Serum TSH levels were analyzed in the central laboratory of the University Medicine Greifswald by a homogeneous, sequential, chemiluminescent immunoassay based on LOCI technology (Dimension Vista System Flex reagent cartridge, Siemens Healthcare Diagnostics Inc., Newark, DE, USA). The analytical measuring range was 0.005–100 mIU/mL and the functional sensitivity was 0.005 mIU/L. High and low TSH were defined using the reference limits 0.49 mIU/L and 3.29 mIU/L as established in SHIP-Trend-0^23^. Further, we calculated structural parameters of the thyroid hormone feedback loop including thyroid's secretory capacity (SPINA-GT), the sum activity of 5′-deiodinases (SPINA-GD) and Jostel’s TSH as a measure for the central set point of the feedback loop, which can be interpreted as a fT4-adjusted TSH level^19,31^.

### Statistical methods

Stratified by thyroid function status, continuous data is presented as median, 25th, and 75th percentile and categorical data as absolute numbers and percentages. Linear regression models adjusted for age, sex, smoking status, and study were used to associate serum TSH levels with markers of body composition. For each marker of body composition we calculated three models—the first using TSH as continuous variable over the full range, the second categorizing TSH into the three groups high, low, and TSH in the reference range, and the third using TSH as continuous variable over the reference range under exclusion of individuals with high or low TSH. In these analyses a *p* < 0.05 was considered as statistically significant. To investigate potential effect modification by age and sex, we introduced interactions of TSH with age and sex in the liner regression models. Here a *p* < 0.1 was considered as statistically significant. All analyses were conducted with Stata 16.1 (Stata Corporation, College Station, TX, USA).

### Ethics approval and consent to participate

All participants gave informed written consent and both studies followed the recommendations of the Declaration of Helsinki and were approved by the Ethics Committee of the University of Greifswald.

## Supplementary Information

Supplementary Information.

## Data Availability

Data from the "Study of Health of Pomerania" are available from the University Medicine Greifswald, Germany but restrictions apply to the availability of these data, which were used under license for the current study, and so are not publicly available. Data are however available upon reasonable request at https://www.fvcm.med.uni-greifswald.de/dd_service/data_use_intro.php and with permission of the University Medicine Greifswald.
