# Supplementary material for: Low serum TSH levels are associated with low values of fat-free mass and body cell mass in the elderly
Source: Sci Rep. 2021 May 18;11:10547. doi: 10.1038/s41598-021-90178-7 (PMC8131378; doi:10.1038/s41598-021-90178-7)
Supplement: Supplementary file 1 — Supplementary Information. [file 41598_2021_90178_MOESM1_ESM.docx]

**Supplementary Table 1.** Associations between serum TSH levels and indices of body composition

|  | TSH; mIU/L | 0.49≤TSH<3.29  β (95%-CI) | TSH<0.49  β (95%-CI) | TSH≥3.29  β (95%-CI) |
| --- | --- | --- | --- | --- |
| Fat mass index; kg/m^2^ | 0.12 (0.05; 0.19)* | Reference | -0.18 (-0.49; 0.11) | 0.01 (-0.47; 0.48) |
| Fat-free mass index; kg/m^2^ | 0.05 (-0.01; 0.10) | Reference | -0.12 (-0.34; 0.10) | -0.16 (-0.50; 0.18) |
| Body cell mass index; kg/m^2^ | 0.01 (-0.02; 0.05) | Reference | -0.12 (-0.26; 0.01) | -0.23 (-0.45; -0.01)* |

Association between TSH and indices of body composition were analyzed by linear regression models adjusted for age, sex, smoking status and study. We calculated two models for each outcome – the first with continuous TSH and the second with TSH categorized into three groups with 0.49≤TSH<3.29 as reference category.

CI confidence interval

*p<0.05

**Supplementary Table 2. Interactions of SPINA-GD and Jostel’s TSH index (TSHI) with age on markers of body composition**

|  | Interaction age and SPINA-GD  p | Interaction age and TSHI  p |
| --- | --- | --- |
| Body mass index; kg/m^2^ | 0.055 | 0.002 |
| Waist circumference; cm | 0.251 | 0.001 |
| Fat mass; kg | 0.397 | 0.341 |
| Fat-free mass; kg | 0.530 | <0.001 |
| Body cell mass (BCM); kg | 0.013 | <0.001 |

p values derived from linear regression models adjusted age, sex, smoking status, study and the respective interaction term
